# Activation of PI3K/AKT/mTOR signaling axis by UBE2S inhibits autophagy leading to cisplatin resistance in ovarian cancer

**DOI:** 10.1186/s13048-023-01314-y

**Published:** 2023-12-19

**Authors:** Mengjun Zhang, Jialin Wang, Yan Guo, Haodi Yue, Lindong Zhang

**Affiliations:** 1 Department of Gynecology, The Third Affiliated Hospital of Zhengzhou University, 7 Rehabilitation Front Street, Zhengzhou, 450052 China; 2 Department of Orthopedics, Xuanwu Hospital, Capital Medical University, Beijing, 100000 China; 3 Department of Oncology, Henan Provincial People’s Hospital, People’s Hospital of Zhengzhou University, No. 7 Weiwu Street, Zhengzhou, 450003 China; 4 Department of Center for Clinical Single Cell Biomedicine, Henan Provincial People’s Hospital, People’s Hospital of Zhengzhou University, No. 7 Weiwu Street, Zhengzhou, 450003 China

**Keywords:** Epithelial OC, Platinum resistance, Ubiquitin-conjugating enzyme E2S, Autophagy

## Abstract

**Background:**

Epithelial ovarian cancer (OC) is the fourth leading cause of cancer-related deaths in women, with a 5-year survival rate of 30%-50%. Platinum resistance is the chief culprit for the high recurrence and mortality rates. Several studies confirm that the metabolic regulation of ubiquitinating enzymes plays a vital role in platinum resistance in OC.

**Methods:**

In this study, we selected ubiquitin-conjugating enzyme E2S (UBE2S) as the candidate gene for validation. The levels of UBE2S expression were investigated using TCGA, GTEx, UALCAN, and HPA databases. In addition, the correlation between UBE2S and platinum resistance in OC was analyzed using data from TCGA. Cisplatin-resistant OC cell lines were generated and UBE2S was knocked down; the transfection efficiency was verified. Subsequently, the effects of knockdown of UBE2S on the proliferation and migration of cisplatin-resistant OC cells were examined through the CCK8, Ki-67 immunofluorescence, clone formation, wound healing, and transwell assays. In addition, the UBE2S gene was also validated in vivo by xenograft models in nude mice. Finally, the relationship between the UBE2S gene and autophagy and the possible underlying regulatory mechanism was preliminarily investigated through MDC and GFP-LC3-B autophagy detection and western blotting experiments. Most importantly, experimental validation of mTOR agonist reversion (the rescuse experiments) was also performed.

**Results:**

UBE2S was highly expressed in OC at both nucleic acid and protein levels. The results of immunohistochemistry showed that the level of UBE2S expression in platinum-resistant samples was significantly higher relative to the platinum-sensitive samples. By cell transfection experiments, knocking down of the UBE2S gene was found to inhibit the proliferation and migration of cisplatin-resistant OC cells. Moreover, the UBE2S gene could inhibit autophagy by activating the PI3K/AKT/mTOR signaling pathway to induce cisplatin resistance in OC in vivo and in vitro.

**Conclusion:**

In conclusion, we discovered a novel oncogene, UBE2S, which was associated with platinum response in OC, and examined its key role through bioinformatics and preliminary experiments. The findings may open up a new avenue for the evaluation and treatment of OC patients at high risk of cisplatin resistance.

**Supplementary Information:**

The online version contains supplementary material available at 10.1186/s13048-023-01314-y.

## Introduction

Epithelial ovarian cancer (OC) is the fourth leading cause of cancer-related deaths in women, with a 5-year survival rate of 30%-50% and platinum resistance is responsible for the high recurrence and mortality rates [1]. However, platinum drugs are at the heart of chemotherapy for OC [2]. Although most patients may initially relapse as they are platinum-sensitive, some eventually become resistant or refractory to platinum chemotherapy [3]. Therefore, in addition to developing new therapeutic options, it is important to examine the unknown mechanisms underlying platinum resistance in OC to improve survival outcomes among drug-resistant patients. In the past decade, the investigation on platinum resistance-related genes has gained traction, however, it is important to continue deeper and more comprehensive evaluation of new biomarkers and their molecular mechanisms of action.

Autophagy and the ubiquitin–proteasome system are the two major intracellular protein degradation pathways that simultaneously regulate important biological processes, including the cell cycle, DNA repair, and apoptosis [4]. The two major protein degradation systems controlled in tandem by the ubiquitin-activating enzyme E1, ubiquitin-binding enzyme E2, and ubiquitin-protein ligase E3 are indispensable in platinum resistance in OC [5]. One of these, namely the ubiquitin-binding enzyme E2S (UBE2S), comprises more than 40 E2 enzymes in human cells. UBE2S is overexpressed in neurological, urological, gastrointestinal, and female genital tumors. Notably, UBE2S regulates DNA repair and is implicated in multiple forms of resistance to chemotherapy in glioblastoma [6–10]. Additional studies show that UBE2S expression is strongly associated with malignancy and resistance to chemoradiation in glioma, thus, an important biomarker of a poor prognosis [11]. The role and mechanism underlying UBE2S function in platinum-based resistance in OC remain unclear, thus arousing our research interest.

Owing to the information explosion and big data, we utilized bioinformatic approaches to mine the genomic datasets for examining the etiology and molecular mechanisms underlying platinum resistance in OC, screening new biomarkers in an effect to understand how platinum resistance evolves in OC, and guide diagnosis, assessment of prognosis, and develop molecular targeted therapies for platinum-resistant OC [12]. Data on platinum resistance in OC from the GEO and TCGA databases were used to screen and evaluate the differentially expressed genes. Survival analysis was performed to optimize differential gene screening. Additionally, we confirmed and examined the above findings in-depth through basic experiments.

To date, there are few reports on the role of ubiquitination-conjugating enzymes in the biology of platinum resistance in OC. Therefore, this study is the first to propose the relevance and potential molecular mechanisms underlying the ubiquitination-binding enzyme, UBE2S in cisplatin resistance in OC. The expression of the UBE2S gene was determined to screen the groups resistant to platinum-based drug therapy or targeted therapy against this gene, which had implications for reversing platinum resistance in OC patients. Therefore, herein, the discovery of the platinum resistance-associated oncogene, UBE2S, is significant for OC patients with platinum resistance, and these findings are expected to open up new therapeutic avenues.

## Materials and methods

### Data collection

At present, there are over 1,000,000 microarray and high-throughput sequencing samples in public databases, including in the Gene Expression Omnibus (GEO), The Cancer Genome Atlas (TCGA), and The Genotype-Tissue Expression datasets. The mRNA expression matrix and patient clinical information in this study were obtained from the GEO, TCGA, and GTEx databases. The platinum-resistant group was defined with the non-platinum treatment interval of < 6 months. A total of 145 cases in the sensitive group and 12 cases in the resistant group were analyzed (Table S1 lists the detailed clinical information). These data were used to examine the level of UBE2S expression and its relationship with platinum resistance and prognosis. Moreover, the differential gene expression of UBE2S was further validated using four OC datasets in the globally recognized comprehensive gene expression database, GEO (https://www.ncbi.nlm.nih.gov/geo/). The four datasets were GSE38666 (tumor = 25, normal = 20), GSE40595 (tumor = 63, normal = 14), GSE18521 (tumor = 66, normal = 9), and GSE26712 (tumor = 185, normal = 10).

### Bioinformatic analyses of UBE2S

First, after the identification of UBE2S as the target gene, the mRNA and protein levels of UBE2S were found to be differential in OC versus normal ovarian tissues in online databases of The Gene Expression Profiling Interactive Analysis (GEPIA, http://gepia.cancer-pku.cn) and UALCAN (http://ualcan.path.uab.edu/index.html). The Kaplan–Meier plotter (http://kmplot.com/analysis/index) was also used to evaluate the effect of the differential levels of expression on the survival outcome of patients. Second, The Human Protein Atlas (http://www.prote inatlas.org), dedicated to human medical oncology research through the mapping of tissues, cells, and organs based on human cancer proteomics, was used. It has a large number of genetic immunohistochemistry results that were used to study the differential protein expression in the tumor and corresponding normal tissues. In the present study, this database was used to determine the differences in levels of UBE2S protein between OC and normal ovarian tissues.

### Patients and clinical tissue samples

This study obtained 63 ovarian cancer tissues from patients who underwent surgery in Zhengzhou University People's Hospital from May 2020 to July 2022. Inclusion criteria: 1) clear pathological diagnosis, 2) complete clinical data, 3) platinum-based chemotherapy after surgery. Exclusion criteria: 1) pathological diagnosis was unclear, 2) patients who had undergone preoperative treatment. Staging was performed according to the International Federation of Gynaecology and Obstetrics (FIGO) system revised in 2009. The study was approved by the Ethics Committee of Zhengzhou University (2023–054).

### Cell culture and construction of resistant strains

The human ovarian cancer cell lines SKOV3 and A2780 were obtained from the Cell Bank of Chinese Academy of Sciences (Shanghai, China). Cells were cultured in 1640 (Corning, Shanghai, China) with 10% fetal bovine serum (FBS; Gibco, Shanghai, China) and penicillin–streptomycin at 37 C with 5% CO2. In this experiment, the concentration gradient culture method was used to construct cisplatin-resistant ovarian cancer cell lines: A2780/DDP and SKOV3/DDP. Cisplatin (DDP) was added to the culture medium of A2780 and SKOV3 human ovarian cancer cells at an initial low concentration of 0.1 µg/mL. After adding cisplatin, wait for the cells to grow stably before passage. The drug concentration of cisplatin was increased every 3 weeks, so that the concentration of cisplatin was increased to 0.2 µg/mL, 0.4 µg/mL, 0.6 µg/mL, 0.8 µg/mL and 1.0 µg/mL. Ovarian cancer cell lines will be transformed into cisplatin-resistant ovarian cancer cell lines with the increase of cisplatin concentration. In this experiment, it was found that the maximum concentration of cisplatin in A2780-DDP cells reached 1 µg/mL, and the cells were in good condition. The maximum concentration of cisplatin in SKOV3-DDP cells reached 2 µg/mL, and the IC50 of cisplatin was determined by CCK8. Cisplatin-resistant ovarian cancer cell lines (A2780/DDP and SKOV3/DDP) were obtained.

### RT-qPCR assay

Total RNA was isolated from SKOV3/DDP and A2780/DDP after cell transfection using TRIzol® (Invitrogen; Thermo Fisher Scientific). Then, RNA solubility was determined by NanoDrop One spectrophotometer (Thermo Fisher Scientific) and reverse transcribed to obtain cDNA (Novoprotein). Finally, the expression level of UBE2S was determined by RT-qPCR using NovoStart SYBR qPCR SuperMix Plus (Novoprotein). The primer sequences of GAPDH and UBE2S were as follows: (GAPDH-F: 5'-CGATGGCATCAAGGTCTTTCCC-3', GAPDH-R: 5'- CAGCAGGAGTTTCATGCGGAAC-3', UBE2S-F: 5'- TCCAAAAATCAAGTGGGGCGA-3', UBE2S-R: 5'- TGATGACCCTTTTGGCTCCC-3'). The thermal cycling conditions were as follows: Initial denaturation at 95 °C for 10 min, denaturation at 95 °C for 10 s, annealing and extension at 60 °C for 30 s, for a total of 40 cycles.

### Western blotting

The corresponding experimental cells were lysed with 1% protease inhibitor. The supernatant was collected by centrifugation at 12,000 g for 15 min at 4ºC. Quantification of protein concentration was performed according to the BCA protein quantification kit. After separation by 10% SDS-PAGE gel electrophoresis, the cells were transferred to PVDF membrane and sealed with blocking solution for 1.5 h. Membranes were incubated with primary antibody UBE2S (Absin, China) overnight at 4 °C. After washing the membrane, horseradish peroxidase-conjugated rabbit secondary antibody was added and incubated at room temperature for 1 h. After that, after washing the membrane for 3 times, the target protein was developed with ECL supersensitive chromogenic solution, and the gel imaging system was used for photographing and analysis. The mTOR pathway activator (MHY1485) was added to the cells after UBE2S knockdown, and Western blot was performed to detect the expression of autophagy-related proteins.

### Immunohistochemistry

Tissue blocks were cut into 4-μm thick sections and stained with hematoxylin. Tissue sections were sequentially deparaffinized, hydrated, and antigen retrieved. After washing with PBS, anti-UBE2S (1:100; Absin, China) was added and incubated overnight at 4 °C. Specimens were washed with PBS and then incubated with horseradish peroxidase secondary antibody and DAB for 45 min at room temperature. After 10 min of color development, counterstain with hematoxylin for 2 min. After the final dehydration, the slides were dried and observed under a microscope, photographed and scored. UBE2S protein expression levels were semi-quantitatively classified based on a total combined score of percentage of positively stained tumor cells and staining intensity. Staining intensity was graded as follows: 0 (no staining); 1 (weak staining); 2 (moderate staining); and 3 (strong staining). Percent staining was scored as 0 (< 5% positive cells), 1 (5%-25% positive cells), 2 (25%-50% positive cells), 3 (50%-75% positive cells), and 4 (> 75% positive cells) % positive cells). Finally, the percentage of positive cells and the intensity score were added to give the final UBE2S expression score on a scale of 0 to 7. In the current study, a staining index of 4 or higher was defined as the optimal cutoff for high expression. The scoring procedure was performed independently twice by two pathologists experienced in evaluating IHC and unaware of clinicopathological information.

### Cell counting kit-8 (CCK-8) assay

Cells in logarithmic growth phase were collected and digested with trypsin to prepare cell suspension. Cells were seeded in 96-well plates at a density of 1 × 103 cells/well. Cell proliferation rates were measured at 0, 24, 48 and 72 h. Add CCK-8 reagent after co-incubation in the incubator for 2 h before assay. Cell proliferation was determined using a microplate reader (Thermo Scientific, Shanghai, China) at a wavelength of 450 nm.

### Clone formation assay

Four groups of cells were cultured at 200 cells/mL per well in 1640 medium containing 2 μg/mL cisplatin and 10% FBS with 3 replicates per well. The medium was changed every three days and colony formation was observed under the microscope every 7 days. After 10 days, they were fixed with 4% paraformaldehyde for 30 min, stained with 0.1% crystal violet for 10 min, washed gently with ddH2O, and photographed under a microscope for statistical analysis after ventilation and drying.

### Knockdown studies

Cells were seeded into 6-well culture plates at a density of 4 × 10^5^ cells/well. When the cell density reached 70%, A2780/DDP and Skov3/DDP cells were transfected with sh-UBE2S and sh-NC. Supplementary Table 2 shows the sequences of UBE2S-shRNA and sh-NC. Add the transfection reagent to the six-well plate according to the manufacturer's instructions. 24 h after transfection, the transfection rate of cells was detected by RT-qPCR and Western blot, and a series of subsequent experiments were carried out after knockdown.

### Monodansylcadaverine (MDC) assay

Four groups of cells were cultured with 1640 medium and 10% FBS, and each well was seeded at 10,000 cells/mL. MDC staining solution (1 mL, MDC kit, C3018M; Beyoncé, Shanghai, China) was added to each well and incubated for 30 min at 37 °C in a dark cell incubator. Aspirate the MDC staining solution, wash the cells three times with Assay Buffer, and observe the autophagy staining effect under a green fluorescence microscope. The excitation wavelength is 335 nm and the emission wavelength is 512 nm.

### GFP-LC3-B and GFP-P62 single-fluorescent labeling

Cells were cultured at 5 × 105 cells/well in 6-well plates, and 2 mL of complete medium was added to each well. Adenovirus LC3-B was added at 20 MOI/well 4 h after cells adhered. After 24 h of infection, the virus-containing medium was removed and 2 mL of fresh complete medium was added to each well. After 24 h of continuous culture, the cell growth and fluorescent protein levels were observed and photographed and counted.

### Wound healing assay

Seed exponentially growing cells at appropriate densities in 6-well plates. When the cell density reaches 80%, use a pipette (200 µL) to draw a straight line along the center of the plate. The exfoliated cells were gently rinsed with PBS buffer and cultured by adding serum-free medium. The width of the scratches was photographed under the microscope after 0, 24 and 48 h and then analyzed using ImageJ v1.8.0.

### Transwell assay

Cells were seeded at 1 × 105 cells/well in 200 μL of serum-free medium into the upper chamber of a Transwell chamber (Corning, New York, USA). Meanwhile, add 700 µL of medium containing 15% FBS to the lower chamber of the 24-well plate. After 24 h, they were fixed with 4% paraformaldehyde for 20 min and stained with 0.1% crystal violet for 20 min. The non-migrated cells on the upper surface of the chamber were gently wiped off with a cotton swab. Finally, the cells in the lower membrane were photographed with an inverted microscope, and the cells were counted for measurement.

### Ki67 immunofluorescence

After making cell slides, they were fixed in 4% paraformaldehyde for 5 min at room temperature, and washed 3 times with PBS. After washing, cells were blocked with 5% nonfat milk and 0.1% Triton X-100 for 1 h at room temperature, then incubated with anti-Ki67 primary antibody (1:200; Abcam) overnight at 4 °C, followed by labeled secondary antibody (1:500; Sigma-Aldrich) for 1 h and washed with PBS buffer. Nuclei were stained with DAPI for 10 min at 4 °C. Staining was observed under five random fields and statistically analyzed with an immunofluorescence microscope (BX60; Olympus, Tokyo, Japan).

### In vivo tumour xenograft experiments

Ten 4-week-old female severe combined immunodeficient BALB/c nude mice (Beijing Viton Lever Laboratory Animal Technology Co., Ltd.), weighing about 16 ~ 18 g were housed under the IVC independent ventilation system, with attention to strict aseptic feeding and handling. Afterwards, the A2780/DDP cells stably transfected with sh-UBE2S or sh-NC were selected in good growth condition at a density of 5 × 10^5^/µl. The animal operating table was strictly sterilized and the operating instruments and equipment were strictly disinfected and sterilized. The mice were divided into two groups of 5 mice each, and 200 µl of A2780/DDP cells were taken from each group. A2780/DDP cell suspension was injected into the right side of the back of the mice for tumor implantation. The mice were kept under strict control for approximately 2 weeks before the tumors became visible, and weekly measurements were taken to record the weight of the mice and the length (L) and width (W) of the subcutaneous transplanted tumors. The tumors were excised and then the tumor weight g, length L and width W were measured and photographed. Tumor volume was calculated by applying V = L × W^2^ × 0.5. The tumor tissues were preserved in 4% paraformaldehyde and then paraffin-embedded to prepare paraffin sections for subsequent experiments. All animal experiments met ethical standards and were approved by the Animal Ethics Committee of Zhengzhou University (2023–054).

### Statistical analysis

The R software (v.3.6.1 version) was used to perform statistical data analysis. Survival and clinicopathological characteristics data were got from the TCGA database. Then, the overall survival of UBE2S was determined by COX regression and Kaplan–Meier. In addition, Wilcox or Kruskal was used to test the relationship between the expression level of UBE2S and the clinical characteristics.

## Results

### UBE2S is highly expressed in OC and is associated with a poor prognosis

**Fig. 1:**
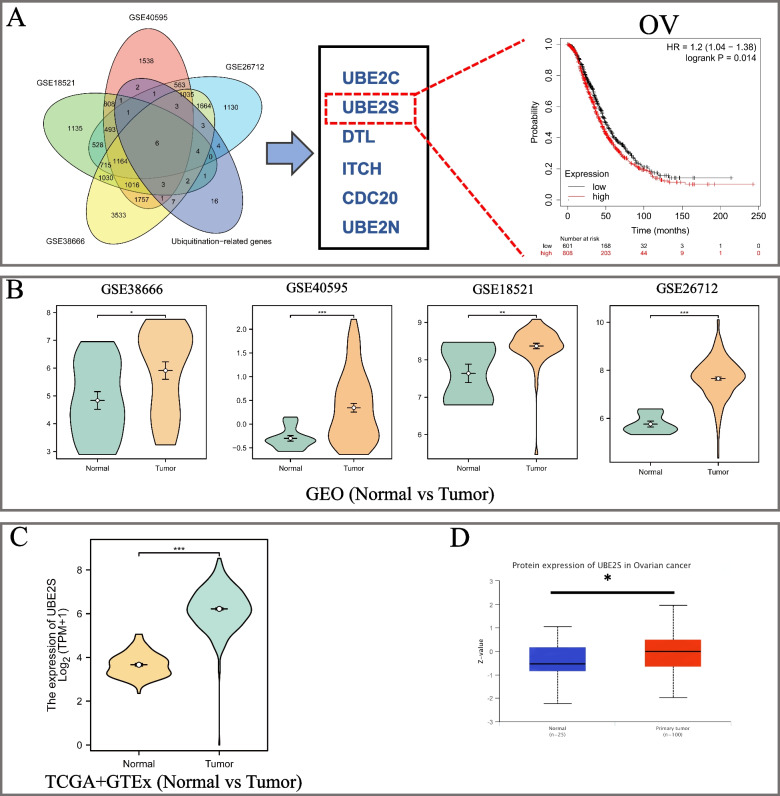
Screening and optimization of functional candidate genes related to ubiquitination of ovarian cancer and the expression level of UBE2S in ovarian cancer. **A** Venn diagrams of four GEO data sets related to ovarian cancer and ubiquitination-related genes (GSE38666, GSE40595, GSE18521, GSE26712). Six candidate genes were screened out (UBE2C, UBE2S, DTL, ITCH, CDC20, UBE2N). The expression level of UBE2S affects the prognosis of patients with ovarian cancer (UBE2S high expression = 388, UBE2S low expression = 169, *p* = 0.0081). **B** A box plot based on the differential expression levels of UBE2S in ovarian cancer and normal ovarian tissues in four GEO data sets. GSE38666, GSE40595, GSE18521, GSE26712. **C** Scatter plots of the differences in the mRNA expression levels of UBE2S in ovarian cancer and normal ovarian tissues in the TCGA dataset and GTEx dataset. **D** A box plot of the differences in the protein expression levels of UBE2S in ovarian cancer and normal ovarian tissues in the UALCAN database (Tumor = 100, Normal = 25). (*p < 0.05, ** *p* < 0.01, *** *p* < 0.001)

**Fig. 2:**
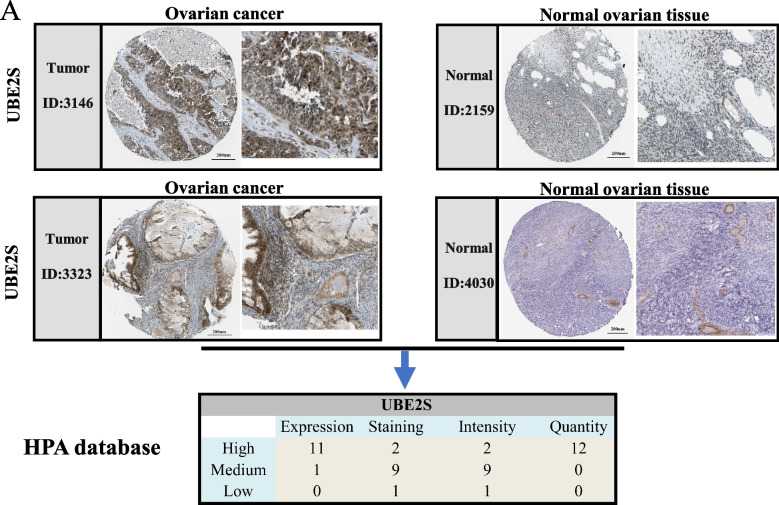
The protein expression of UBE2S gene in ovarian cancer tissues. **A** In the HPA database, the immunohistochemical expression of UBE2S in ovarian cancer (ID: 3146, ID: 3323) showed strong staining. The immunohistochemical expression of UBE2S in normal ovarian tissue (ID: 2159, ID: 4030) showed low staining

A comprehensive understanding of the essential role of ubiquitination-conjugating enzymes in OC in core-linked positions was gained. First, using the intersection of the four OC datasets (GSE38666, GSE40595, GSE18521, and GSE26712) and ubiquitin-conjugating enzyme-related gene sets from GEO, six differential genes (UBE2C, UBE2S, DTL, ITCH, CDC20, UBE2N) were identified (Fig. 1A). Subsequently, further screening was performed using the GEPIA and KM-plotter online databases (Fig. 1A). Finally, screening and optimization suggested that UBE2S may play an important role in OC and has yet not been studied. Therefore, we conducted in-depth analyses with UBE2S as the target gene. After identifying target genes, their role in OC needs to be verified from different perspectives. First, in the four OC-GEO datasets (GSE38666, GSE40595, GSE18521, and GSE26712), UBE2S was found to be highly expressed in tumor tissues as compared to the normal ovarian tissues (Fig. 1B). Moreover, 427 cancerous and 88 normal ovarian tissues in TCGA and GTEx showed abnormally high expression of UBE2S in tumors, consistent with the results from the GEO dataset (Fig. 1C). In addition, in cancer tissues, UBE2S exhibited abnormally high expression not only at the mRNA level but also at the protein level. Moreover, using the HPA database, we found that UBE2S depicted strong staining in the 12 OC tissues, which further confirmed its abnormally high protein expression in cancer tissues (Fig. 2). Taken together, we found abnormally high expression of UBE2S in OC through bioinformatic analyses of high-throughput data, however, experimental verification was further warranted.

### UBE2S is closely associated with platinum resistance in OC

**Fig. 3:**
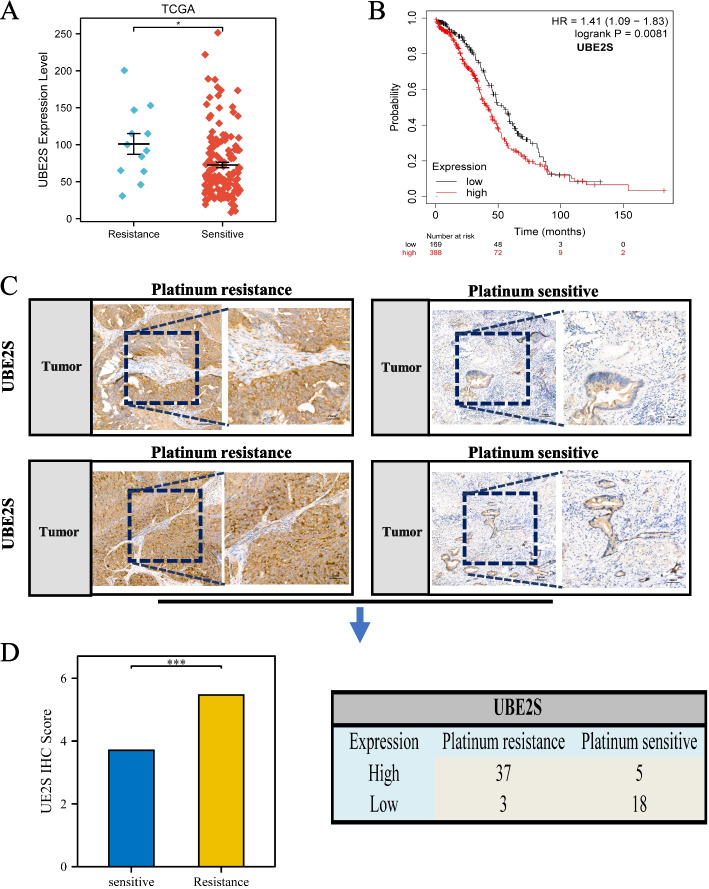
Correlation of UBE2S with platinum resistance in ovarian cancer. **A** A scatter plot of the difference in the expression level of UBE2S in platinum-sensitive and platinum-resistant tissues of ovarian cancer in the TCGA data set. **B** Based on the KM database, the overall survival curve of patients in the low-expression group and high-expression group of UBE2S gene in platinum-resistant chemotherapy patients. The blue line indicates platinum resistance, and the red line indicates platinum sensitivity. **C** The immunohistochemical staining results of 63 patients with ovarian cancer were collected clinically. The fields of view are 100 × and 200 × respectively. **D** Histogram of immunohistochemical scores and the statistical table of UBE2S staining expression of patients

The poor prognosis of OC has been largely attributed to resistance to chemotherapy. The valuable role of ubiquitination in the development of platinum resistance in OC has been well established. As shown in Fig. 3A, the platinum-resistant group (*n* = 145) showed abnormally high expression of UBE2S as compared to the sensitive group (*n* = 12), according to the interval of platinum treatment. Subsequently, the intersection of GEO datasets (GSE41499, GSE15373, GSE14764, and GSE14231) associated with platinum resistance in OC yielded a total of 83 differential genes; among them, 29 were significantly associated with UBE2S (Fig. S2). This aroused our interest in further examining the correlation and mechanism of action of UBE2S in platinum resistance in OC. Next, survival analysis suggested that high expression of UBE2S in platinum-treated patients resulted in a poor prognosis (Fig. 3B). Finally, in the context of our bioinformatic analyses, UBE2S was found to play an important role in platinum resistance in OC. Therefore, we used the OC clinical tissues obtained from 63 patients for experimental validation. Immunohistochemistry of paraffin-embedded OC sections from 63 patients suggested that UBE2S showed an abnormally high expression in 37 platinum-resistant and 5 platinum-sensitive patients (Fig. 3C-D). The expression of UBE2S protein in platinum-resistant patients was markedly increased, *p* < 0.05. Thus, the high gene expression of UBE2S was closely related to the drug resistance in OC patients.

### Knocking down UBE2S inhibits proliferation and migration of cisplatin-resistant OC cells

**Fig. 4:**
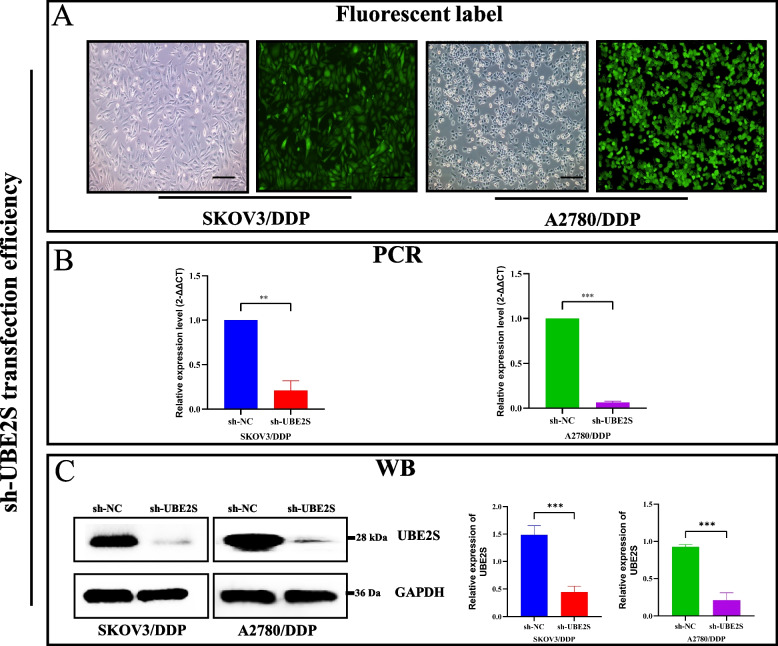
Validation of sh-UBE2S gene transfection efficiency in cisplatin-resistant cell lines. **A** Twenty-four hours after lentiviral transfection of Sh-UBE2S, the transfection efficiency was observed by fluorescence microscopy. Green fluorescence indicates successful transfection. **B** PCR-validated histogram of changes in gene expression levels following lentiviral transfection of Sh-UBE2S. Statistical analysis was performed using 2-∆∆Ct values. RT-qPCR was used to detect the transfection efficiency of UBE2S. Data are presented as mean ± standard deviation (SD). **C** Western blotting experiments verifying the changes in gene protein levels after lentiviral transfection of Sh-UBE2S and histograms of the results of statistical analysis (*p < 0.05, **p < 0.01, ***p < 0.001)

According to the experimental results, UBE2S was found to be overexpressed in cisplatin-resistant cancers. Therefore, we constructed cisplatin-resistant OC cell lines (Fig. S3) and knocked down UBE2S; the transfection efficiency was validated. Fluorescence microscopy images showed that in the SKOV3/DDP and A2780/DDP cells, those with green GFP fluorescence labeling in the sh-UBE2S group accounted for more than 90% of the total number of cells (Fig. 4A), thereby suggesting that the transfection efficiency was sufficient. The results of RT-qPCR and western blotting (Fig. 4B-C) showed that in the SKOV3/DDP and A2780/DDP cells, the relative mRNA and protein expressions of UBE2S in the sh-UBE2S group were significantly lower relative to the sh-NC group. Therefore, the results of immunofluorescence, RT-qPCR, and western blotting confirmed the knockdown efficiency of the UBE2S gene in the cisplatin-resistant OC cell lines to be sufficient.

**Fig. 5:**
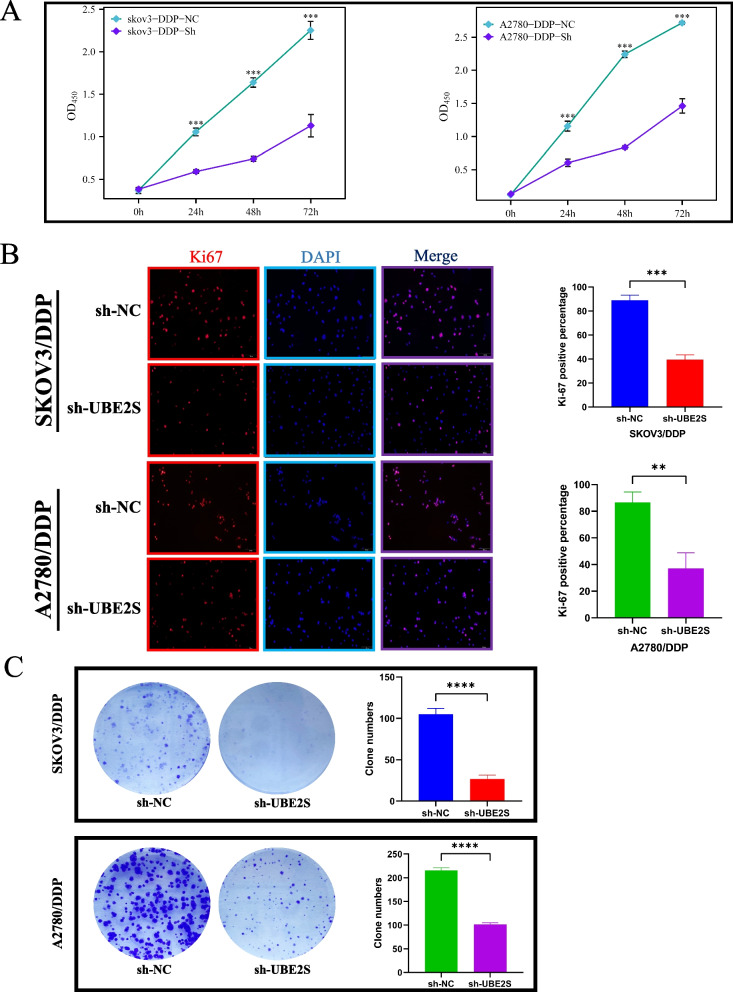
The effect of UBE2S gene knockdown on the proliferation of platinum-resistant ovarian cancer cells. **A** Statistical analysis of CCK8 test results of SKOV3/DDP and A2780/DDP cell lines. The blue line represents the control group, and the yellow line represents the UBE2S gene knockdown group. The cell proliferation rate was measured at 0 h, 24 h, 48 h and 72 h. **B** Ki-67 immunofluorescence staining of the control group and UBE2S gene knockdown group in SKOV3/DDP and A2780/DDP cell lines. Red is Ki-67 staining, blue is DAPI nuclear staining, and Merge merges the image. The histogram counts the number of Ki-67 cells. **C** Experimental results and statistics of cell colony formation after SKOV3/DDP and A2780/DDP cell line transfection. *p < 0.05, **p < 0.01, ***p < 0.001

Next, the proliferation ability of cisplatin-resistant OC cells was evaluated using the CCK-8, Ki-67 immunofluorescence, and clone formation assays. As shown in Fig. 5A, in the SKOV3/DDP and A2780/DDP cells, the absorbance (OD values) in the sh-UBE2S group at 24, 48, and 72 h after cell transfection were significantly lower than those in the sh-NC group, suggesting that the UBE2S knockdown resulted in a decreased cell proliferation activity. As shown in Fig. 5B, in SKOV3/DDP and A2780/DDP cells, the percentage of Ki-67 positive cells in the sh-UBE2S group was significantly lower relative to the sh-NC group, suggesting that the number of cells in the proliferation phase in the UBE2S knockdown group reduced significantly. As shown in Fig. 5C, in both SKOV3/DDP and A2780/DDP cells, the number of colonies formed in the sh-UBE2S group was significantly lower as compared to the sh-NC group, suggesting that the UBE2S knockdown group had a reduced clone forming ability. Therefore, the results of the CCK-8, Ki-67 immunofluorescence, and clone formation assays suggested that knocking down UBE2S could substantially inhibit the proliferation of cisplatin-resistant OC cells.

**Fig. 6:**
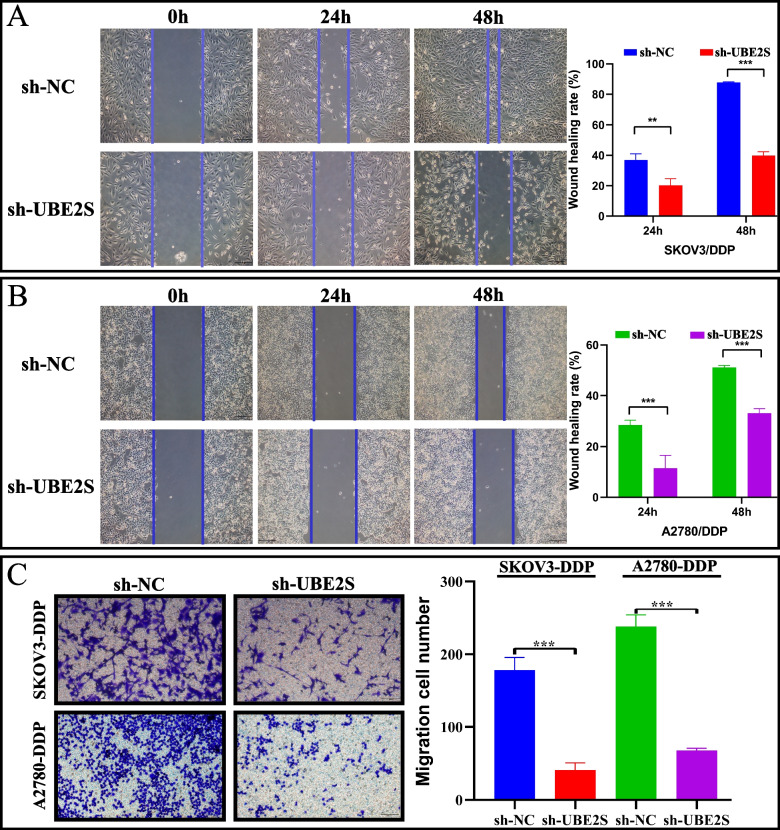
The effect of UBE2S gene knockdown on the migration of cisplatin-resistant ovarian cancer cells. **A** Wound healing experiment and analysis results of SKOV3-DDP cell line. The cell migration rate was measured at 0 h, 24 h and 48 h. **B** Wound healing experiment and analysis results of A2780-DDP cell line. The cell migration rate was measured at 0 h, 24 h and 48 h. The histogram shows the migration rate (*p < 0.05, **p < 0.01, ***p < 0.001). **C** The migration ability of A2780-DDP and SKOV3-DDP cell lines was determined by Transwell experiment and comparative analysis results

Finally, the migration ability of cisplatin-resistant OC cells was evaluated using the cell scratch and Transwell assays. Figure 6A-B show that in the SKOV3/DDP and A2780/DDP cells, the scratch healing rate in the sh-UBE2S group at 24 and 48 h after cell transfection was significantly lower relative to the sh-NC group, suggesting a decreased cell migration ability in the UBE2S knockdown group. Figure 6C shows that in both SKOV3/DDP and A2780/DDP cells, the number of cells that migrated to the lower chamber of the Transwell in the sh-UBE2S group was significantly lower as compared to that in the sh-NC group, suggesting the reduced cell migration ability in the UBE2S knockdown group. Therefore, the results of cell scratch and Transwell assays suggested that the gene knockdown of UBE2S significantly inhibited the migration ability of cisplatin-resistant OC cells in both migration distance and cell migration number.

Therefore, the results of CCK-8, Ki-67 immunofluorescence, clone formation, cell scratch, and Transwell assays together suggested that gene knockdown of UBE2S could inhibit the proliferation and migration of cisplatin-resistant OC cells. In combination with the abnormally high expression of UBE2S in cisplatin-resistant OC as described above, we reasonably speculated that the abnormally high expression of UBE2S may promote drug resistance and malignancy in OC by enhancing the proliferation and migration ability of cisplatin-resistant OC cells.

### Knocking down UBE2S activates autophagy in cisplatin-resistant OC cells

**Fig. 7:**
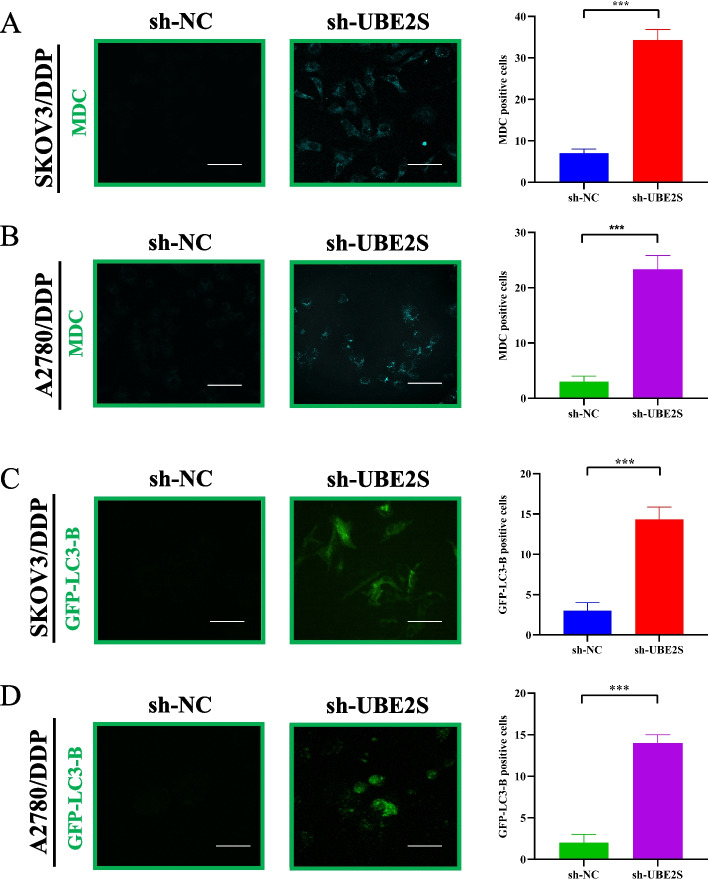
The relationship between UBE2S gene knockout and autophagy in cisplatin-resistant ovarian cancer. **A** MDC-stained confocal micrographs of autophagy induced by knockdown of UBE2S in SKOV3/DDP cells. **B** MDC-stained confocal micrographs of autophagy induced by knockdown of UBE2S in A2780/DDP cells. MDC immunofluorescence staining of sh-NC and sh-UBE2S-treated cells for 24 h. Green is MDC-stained autophagosomes. **C** GFP-LC3-B adenovirus immunofluorescence marker staining in SKOV3/DDP cell lines in sh-NC and sh-UBE2S-treated groups. **D** GFP-LC3-B adenovirus immunofluorescence marker staining in A2780/DDP cell lines in sh-NC and sh-UBE2S-treated groups. * *p* < 0.05, ** *p* < 0.01, *** *p* < 0.001

**Fig. 8:**
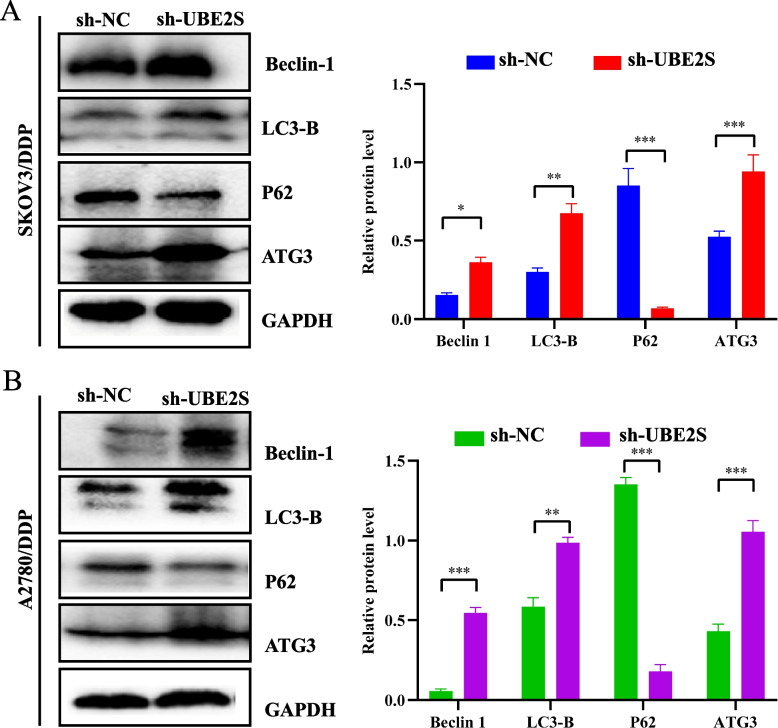
The relationship between UBE2S gene knockout and autophagy-related genes in cisplatin-resistant ovarian cancer. **A** Western blotting results and statistical analysis histograms of Beclin-1, LC3-B, P62 and ATG3 in experimental group and control cells of SKOV3/DDP cell line. **B** Western blotting results and statistical analysis histograms of Beclin-1, LC3-B, P62 and ATG3 in experimental group and control cells of A2780/DDP cell line. *p < 0.05, **p < 0.01, ***p < 0.001

The above results confirmed that UBE2S could promote the malignant progression of OC and the drug resistance phenotype. Given that autophagy is an important mechanism regulating chemoresistance and shows an inseparable relationship with ubiquitination, we, therefore, examined the relationship between UBE2S and autophagy in cisplatin-resistant OC cells. MDC autophagy assay and GFP-LC3-B adenovirus transfection and immunofluorescence assay were performed to evaluate the changes in autophagy. The results of immunofluorescence staining of MDC and GFP-LC3-B (Fig. 7A-D) showed that the green fluorescence in A2780/DDP and SKOV3/DDP cells was significantly higher relative to the sh-UBE2S group. Finally, the results of western blotting (Fig. 8) showed that in the SKOV3/DDP and A2780/DDP cells, the relative expressions of autophagy signature proteins (ATG3, Beclin-1, and LC3-B) in the sh-UBE2S group were significantly higher relative to the sh-NC group, while P62 protein showed markedly lower expression as compared to the sh-NC group. This suggested that the expression of these autophagy characteristic proteins in cisplatin-resistant OC cells in the UBE2S knockdown group changed significantly. Thus, UBE2S may be involved in inhibiting autophagy in cisplatin-resistant OC cells The regulation of OC could promote its cisplatin resistance and malignant progression.

### UBE2S induces cisplatin resistance in OC by inhibiting impaired autophagy through PI3K/AKT/mTOR activation

**Fig. 9:**
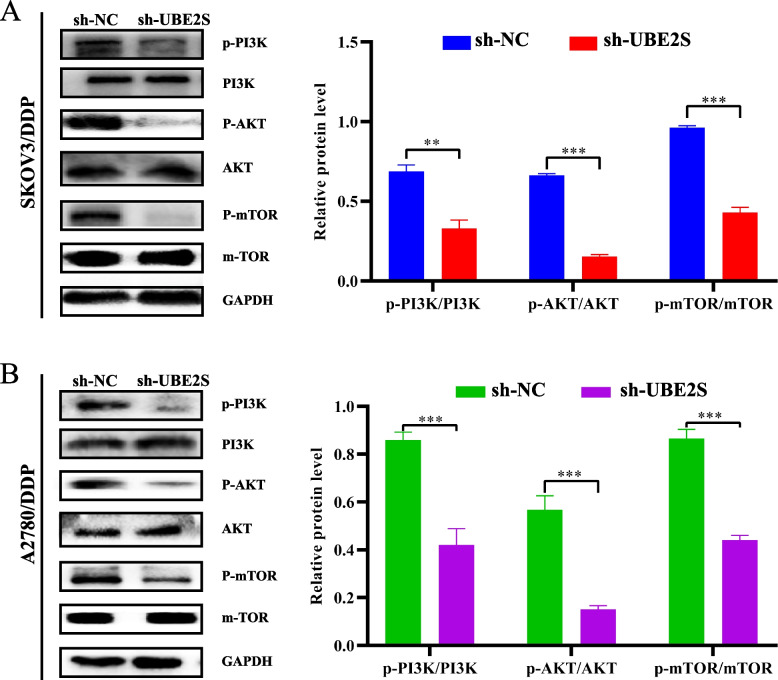
The relationship between UBE2S gene knockout and PI3K/AKT/mTOR signaling pathway-related genes in cisplatin-resistant ovarian cancer. **A** Western blotting results and statistical analysis histogram of p-PI3K, PI3K, p-AKT, AKT, p-mTOR and mTOR in experimental group and control cells of SKOV3/DDP cell line. **B** Western blotting results and statistical analysis histogram of p-PI3K, PI3K, p-AKT, AKT, p-mTOR and mTOR in experimental group and control cells of A2780/DDP cell line. *p < 0.05, **p < 0.01, ***p < 0.001

Given that the PI3K/AKT/mTOR signaling pathway is a key upstream negative regulator of autophagy, we evaluated the relationship between UBE2S and the PI3K/AKT/mTOR signaling pathway. The results of western blotting (Fig. 9) showed that in the SKOV3/DDP and A2780/DDP cells, the relative expression of PI3K, AKT, and mTOR proteins in the sh-UBE2S group was significantly lower as compared to the sh-NC group. The inhibition of the PI3K/AKT/mTOR signaling pathway also suggested that UBE2S may be involved in promoting the PI3K/AKT/mTOR signaling activity in cisplatin-resistant OC cells, thereby inhibiting autophagy and leading to their malignant progression. MHY1485 acts as an activator of the PI3K/AKT/mTOR signaling pathway. As shown in Fig. S2C-D, the expression of autophagy-related proteins increased after UBE2S gene knockdown, but after the addition of mTOR activators (MHY1485), the expression of autophagy proteins that should have been increased due to UBE2S gene knockdown was decreased, that is, autophagy was reversed. This can reconfirm the above conclusion that UBE2S knockdown activates autophagy in OC/DDP cells by inhibiting PI3K/AKT/mTOR signaling pathway.

### Knockdown of UBE2S inhibits the growth of cisplatin-resistant ovarian cancer transplant tumors in mice

**Fig. 10:**
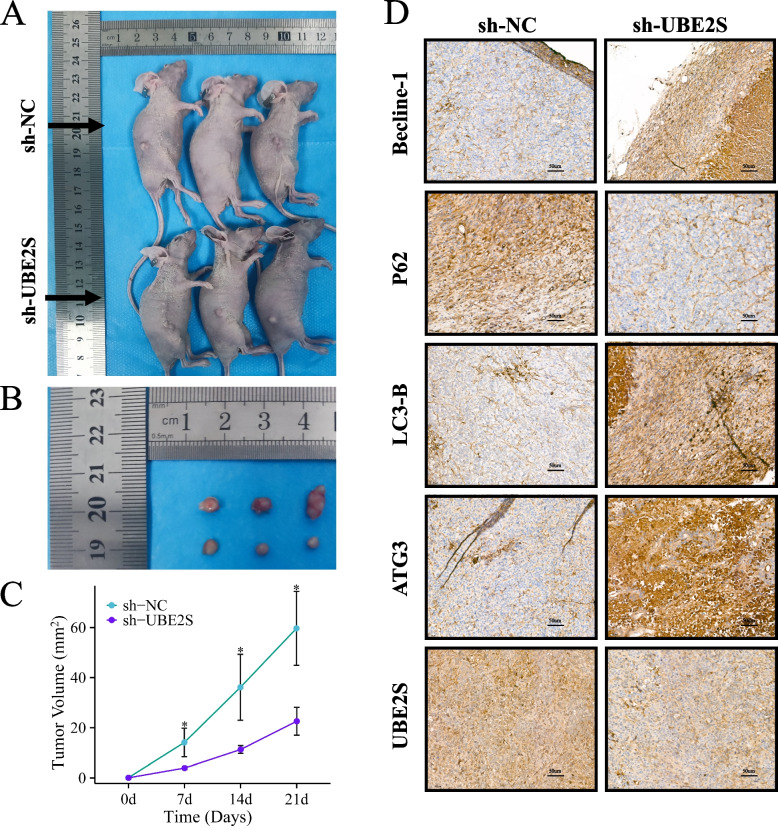
In vivo experiments with the nude mouse xenograft model. **A** A2780/DDP ovarian cancer cells transfected with sh-NC or sh-UBE2S were injected into the abdominal back of BALB/c nude mice. **B** After injection of A2780/DDP cells, the tumor was removed on the 21nd day, and the long diameter (L mm) and wide diameter (W mm) of the tumor were measured **C** Growth curves of BALB/c nude mice grafts transfected with sh-NC or sh-UBE2S, **p < 0.05. **D** Protein expression of autophagy-related genes by immunohistochemistry in tumor tissues of nude mice transplanted with tumors

Figure 10A shows a nude mouse following xenograft transplantation. Figure 10B showed the tumor removed from the abdominal dorsum of the mouse. It could be seen from the images that the transplanted tumor size was smaller in the sh-UBE2S group compared to the sh-NC group. Figure 10B illustrated that by comparing the volume of transplanted tumors removed on day 21 after injection of A2780/DDP cells, it was found that the volume of transplanted tumors was smaller in the sh-UBE2S group compared to the sh-NC group, and the difference was statistically significant. Figure 10C showed the growth curve of mouse transplanted tumors based on the weekly volume of mouse transplanted tumors, which showed the slow growth of transplanted tumors in the sh-NC group compared to the sh-UBE2S group. Moreover, immunohistochemical staining of the removed tumor tissues showed differential expression of autophagy-related proteins in the sh-UBE2S group (increased expression of Becline-1, LC3-B, ATG3; decreased expression of P62), suggesting that the above results indicated that the growth of transplanted tumors in the nude mouse with UBE2S knockdown was affected by the inhibition of autophagy. The growth of transplanted tumors was significantly inhibited after UBE2S knockdown. In vivo experiments demonstrated that UBE2S promoted the growth of cisplatin-resistant ovarian cancer transplant tumors in the nude mouse.

## Discussion

Epithelial OC is a malignant tumor of the female reproductive tract with a 5-year survival rate as low as 30%; platinum resistance has been implicated as one of the main culprits for the high mortality among these patients [13, 14]. Platinum-resistant patients continue to receive non-platinum cytotoxic agents as the standard care, and they often have a poor prognosis. For BRCA mutation-positive patients, a few options for targeted therapy other than PARP inhibitors are available. Treatment options for patients with platinum-resistant OC pose a major challenge for gynecological oncologists [15]. Therefore, identification of promising drug targets is required. However, in addition to the need for in-depth evaluation of new therapeutic targets and understanding of mechanisms underlying chemoresistance, emphasis should be placed on discovering specific new biomarkers to facilitate the early assessment of platinum-resistant high-risk populations and develop preventive and individualized treatment strategies. In this study, first, we bioinformatically screened the differentially expressed genes using GEO and TCGA datasets and obtained the candidate genes closely related to platinum resistance. Next, expression was analyzed and UBE2S levels were validated using a large database. Finally, tissue, cell, and in vivo experiments were performed to confirm the close relationship between UBE2S and platinum resistance and determine the possible molecular mechanism underlying UBE2S-mediated platinum resistance. In combination with bioinformatic high-throughput data analysis and scientific experiments, UBE2S was identified as a biomarker for evaluating OC prognosis and predicting cisplatin resistance, thus implicating it as a potential therapeutic target for reversing platinum resistance.

The ubiquitin system and autophagy are the two major protein degradation systems in the human body. These are essential for many biological processes, including signal transduction, regulation of cell survival, protein degradation, and DNA damage repair. The bridge ubiquitin-conjugating enzyme, UBE2S, of the ubiquitin system has gained attention owing to its abnormally high expression in many human malignant tumors and its close relationship with patients' prognoses and treatment responses. Overexpression of UBE2S promotes tumor cell proliferation and migration by regulating the VHL/HIF-1α/STAT3 signaling pathway [16]. In addition, UBE2S also promotes the development of lung cancer through the canonical Wnt signaling pathway [17]. In clinical settings, UBE2S is known to be an important factor influencing drug treatment sensitivity. For example, UBE2S is used as a new marker to predict the efficacy of radiotherapy and chemotherapy in human glioma. It binds to Ku70 and regulates DNA repair, thereby affecting chemotherapy drug resistance in the multi-form glioblastoma [6]. UBE2S is recruited to the DNA damage sites and is responsible for Lys11-linkage ubiquitin modification of chromatin-binding proteins (including H2A/H2AX), which may be involved in DNA damage repair due to chemotherapeutic drugs, thus affecting the treatment response rates [18]. Despite the role of UBE2S in tumor proliferation, chemoresistance, and metastases, the function of accumulated UBE2S in epithelial OC and its association with platinum resistance remain largely unknown.

In this study, we confirmed the close relationship of UBE2S with platinum resistance and the malignant progression of OC through bioinformatic analyses of big data. First, UBE2S was found to be highly expressed in OC tissues at both mRNA and protein levels and resulted in a poor prognosis. Importantly, higher expression of UBE2S was observed in platinum-resistant OC tissues. Second, UBE2S was closely related to many genes associated with platinum resistance. This suggested that UBE2S may have an unknown close relationship with platinum resistance in OC. Therefore, further studies are necessary to demonstrate the efficacy of UBE2S as a predictive biomarker of platinum resistance and the underlying molecular mechanisms in the progression of platinum resistance.

Autophagy and ubiquitination are turn-key regulators regulating the balance between cell survival and death. On the one hand, autophagy affects the sensitivity of OC cells to cisplatin by controlling endoplasmic reticulum stress through the expression of p62 in cisplatin-resistant OC cells. On the other hand, pathways in autophagy can interact with other switch proteins by combining with polyubiquitin chains, resulting in dual roles of pro-survival or pro-apoptosis [19]. Resistance is the most serious limitation to platinum-based chemotherapy. To improve chemosensitivity and reverse drug resistance, in addition to examining new specific drug resistance-related biomarkers, the relevant molecular mechanisms must be elucidated. The mechanisms of platinum resistance in epithelial OC are in part multifactorial, including decreased intracellular drug accumulation, increased detoxification systems, increased DNA repair processes, and decreased apoptosis, as also autophagy [20].

The ubiquitinase, UBE2S, may affect the platinum resistance in OC by regulating the metabolic system, thereby reducing the accumulation of intracellular drugs, increasing the repair of DNA damage, or promoting autophagy. On the one hand, by knocking down the expression of UBE2S, we found that the loss of UBE2S expression could alter the malignant biological behaviors of platinum-resistant cell lines, including cellular proliferation and migration. On the other hand, after knocking down UBE2S, autophagy was activated as evidenced by the results of the MDC staining and GFP-LC3-B experiments. Therefore, we reasonably speculated that UBE2S could affect the occurrence of platinum resistance in OC through autophagy. Based on cell, tissue and in vivo animal experiments, this was further confirmed by the expressions of autophagy-related genes, ATG5, Beclin-1, and LC3-B in the sh-UBE2S group, which were all increased, while that of P62 reduced markedly. Taken together, UBE2S may induce platinum resistance in OC by inhibiting damaged autophagy.

However, how UBE2S affects autophagy requires further investigation. Mature autophagy-related regulatory pathways include the PI3K-AKT-mTOR and MAPK cascades. The PI3K/AKT/mTOR signaling pathway plays a key role in multiple functions including apoptosis and autophagy [21, 22]. Finally, based on the western blot and rescue experiments in Figs. 9 and S3C-D, UBE2S could induce cisplatin resistance in OC by activating the PI3K/AKT/mTOR signaling pathway and inhibiting impaired autophagy. Interestingly, Rottlerin et al. have shown induction in autophagy by inhibiting the PI3K/AKT/mTOR signaling pathway to promote apoptosis in human pancreatic cancer stem cells [23]. Taken together, the results of western blotting suggested that the UBE2S gene may inhibit damaged autophagy by activating the PI3K/AKT/mTOR signaling pathway to maintain cisplatin resistance in OC cells, ultimately leading to malignant progression and a poor prognosis. UBE2S can be used as a novel biomarker to predict the platinum-resistant population in OC. It affected autophagy through the PI3K/AKT/mTOR signaling pathway and is a potential target of gene therapy and targeted therapy for OC and reversing drug resistance.

In this study, high-throughput big data analyses and solid basic experimental verifications suggested a reliable biomarker closely related to platinum resistance in OC, and an in-depth evaluation and discussion of its role in platinum-resistant OC malignant biological have been presented. Analysis of the characteristics and molecular mechanisms may provide a new perspective for the study of platinum resistance in OC, such as in the identification of a new platinum resistance gene for potential mechanism research or clinical application, as described herein.

## Conclusion

In summary, we identified a novel oncogene, UBE2S, which was associated with platinum-based OC response, and through big data-high-throughput analysis and experimental verification in vivo and in vitro, we examined its roles and molecular mechanisms of action in platinum-resistant OC. We inferred that UBE2S may induce drug resistance by inhibiting impaired autophagy, resulting in poor patient prognoses. The findings not only reveal a new prognostic biomarker for cisplatin resistance in OC but also open up new treatment avenues for cisplatin-resistant OC patients.

### Supplementary Information

**Additional file 1: Table S1.** TCGA Ovarian Cancer Patient Baseline Information.

**Additional file 2: Table S2. **Sequences of UBE2S-shRNA and sh-NC.

**Additional file 3:** **Figure S1.** The main idea and steps of this study.

**Additional file 4:** **Figure S2.** Correlation analysis between UBE2S and platinum resistance-related genes in patients with ovarian cancer.

**Additional file 5:** **Figure S3.** The effect of UBE2S gene knockdown on the sensitivity of ovarian cancer cells to cisplatin treatment and the construction of cisplatin-resistant cell lines.

## Data Availability

The data can be obtained through the email under reasonable request: zld8399@163.com.

## Abbreviations

OC, OV: Ovarian cancer
UBE2S: Ubiquitin-conjugating enzyme E2S
UBE1: Ubiquitin-like modifier activating enzyme 1
UBE3: Ubiquitin-protein ligase E3
TCGA: The Cancer Genome Atlas
GEO: Gene Expression Omnibus
IHC: Immunohistochemistry
