# Supplementary material for: Activation of PI3K/AKT/mTOR signaling axis by UBE2S inhibits autophagy leading to cisplatin resistance in ovarian cancer
Source: J Ovarian Res. 2023 Dec 19;16:240. doi: 10.1186/s13048-023-01314-y (PMC10729389; doi:10.1186/s13048-023-01314-y)
Supplement: Supplementary file 1 — Additional file 1: Table S1. TCGA Ovarian Cancer Patient Baseline Information. [file 13048_2023_1314_MOESM1_ESM.docx]

| **Characteristic** | **Resistance** | **Sensitive** | **p** |
| --- | --- | --- | --- |
| n | 12 | 145 |  |
| Race, n (%) |  |  | 0.849 |
| AMERICAN INDIAN OR ALASKA NATIVE | 0 (0%) | 1 (0.6%) |  |
| ASIAN | 0 (0%) | 2 (1.3%) |  |
| BLACK OR AFRICAN AMERICAN | 0 (0%) | 10 (6.4%) |  |
| Not Available | 0 (0%) | 2 (1.3%) |  |
| WHITE | 12 (7.6%) | 130 (82.8%) |  |
| lymphatic invasion, n (%) |  |  | 0.675 |
| NO | 1 (0.6%) | 13 (8.3%) |  |
| Not Available | 8 (5.1%) | 109 (69.4%) |  |
| YES | 3 (1.9%) | 23 (14.6%) |  |
| Histologic grade, n (%) |  |  | **0.014** |
| G2 | 2 (1.3%) | 22 (14%) |  |
| G3 | 9 (5.7%) | 120 (76.4%) |  |
| G4 | 1 (0.6%) | 0 (0%) |  |
| GB | 0 (0%) | 1 (0.6%) |  |
| GX | 0 (0%) | 2 (1.3%) |  |
| Clinical stage, n (%) |  |  | 0.493 |
| Stage IIB | 0 (0%) | 1 (0.6%) |  |
| Stage IIC | 1 (0.6%) | 2 (1.3%) |  |
| Stage IIIA | 0 (0%) | 4 (2.5%) |  |
| Stage IIIB | 0 (0%) | 8 (5.1%) |  |
| Stage IIIC | 8 (5.1%) | 106 (67.5%) |  |
| Stage IV | 3 (1.9%) | 24 (15.3%) |  |
| Residual disease, n (%) |  |  | 0.608 |
| >20 mm | 1 (0.6%) | 29 (18.5%) |  |
| 1-10 mm | 9 (5.7%) | 83 (52.9%) |  |
| 11-20 mm | 1 (0.6%) | 8 (5.1%) |  |
| No Macroscopic disease | 0 (0%) | 14 (8.9%) |  |
| Not Available | 1 (0.6%) | 11 (7%) |  |
| Venous invasion, n (%) |  |  | 0.270 |
| NO | 1 (0.6%) | 11 (7%) |  |
| Not Available | 9 (5.7%) | 124 (79%) |  |
| YES | 2 (1.3%) | 10 (6.4%) |  |
| Age, mean ± SD | 60.08 ± 12.11 | 60.61 ± 11.27 | 0.878 |

**Table S1. TCGA Ovarian Cancer Patient Baseline Information.**
