# Supplementary material for: Activation of PI3K/AKT/mTOR signaling axis by UBE2S inhibits autophagy leading to cisplatin resistance in ovarian cancer
Source: J Ovarian Res. 2023 Dec 19;16:240. doi: 10.1186/s13048-023-01314-y (PMC10729389; doi:10.1186/s13048-023-01314-y)
Supplement: Supplementary file 2 — Additional file 2: Table S2. Sequences of UBE2S-shRNA and sh-NC. [file 13048_2023_1314_MOESM2_ESM.docx]

| Sh-UBE2S | Sequence (5’-3’) |
| --- | --- |
| shUBE2S-sense | GGGCTCTCTTCCTCCTTCCAC |
| shUBE2S-antisense | GTGGAAGGAGGAAGAGAGCCC |
| shNC-sense | TTCTCCGAACGTGTCACGT |
| shNC- antisense | ACGTGACACGTTCGGAGAA |

**Table S2. Sequences of UBE2S-shRNA and sh-NC.**
