# Supplementary material for: Activation of PI3K/AKT/mTOR signaling axis by UBE2S inhibits autophagy leading to cisplatin resistance in ovarian cancer
Source: J Ovarian Res. 2023 Dec 19;16:240. doi: 10.1186/s13048-023-01314-y (PMC10729389; doi:10.1186/s13048-023-01314-y)
Supplement: Supplementary file 3 — Additional file 3: Figure S1. The main idea and steps of this study. [file 13048_2023_1314_MOESM3_ESM.pdf]

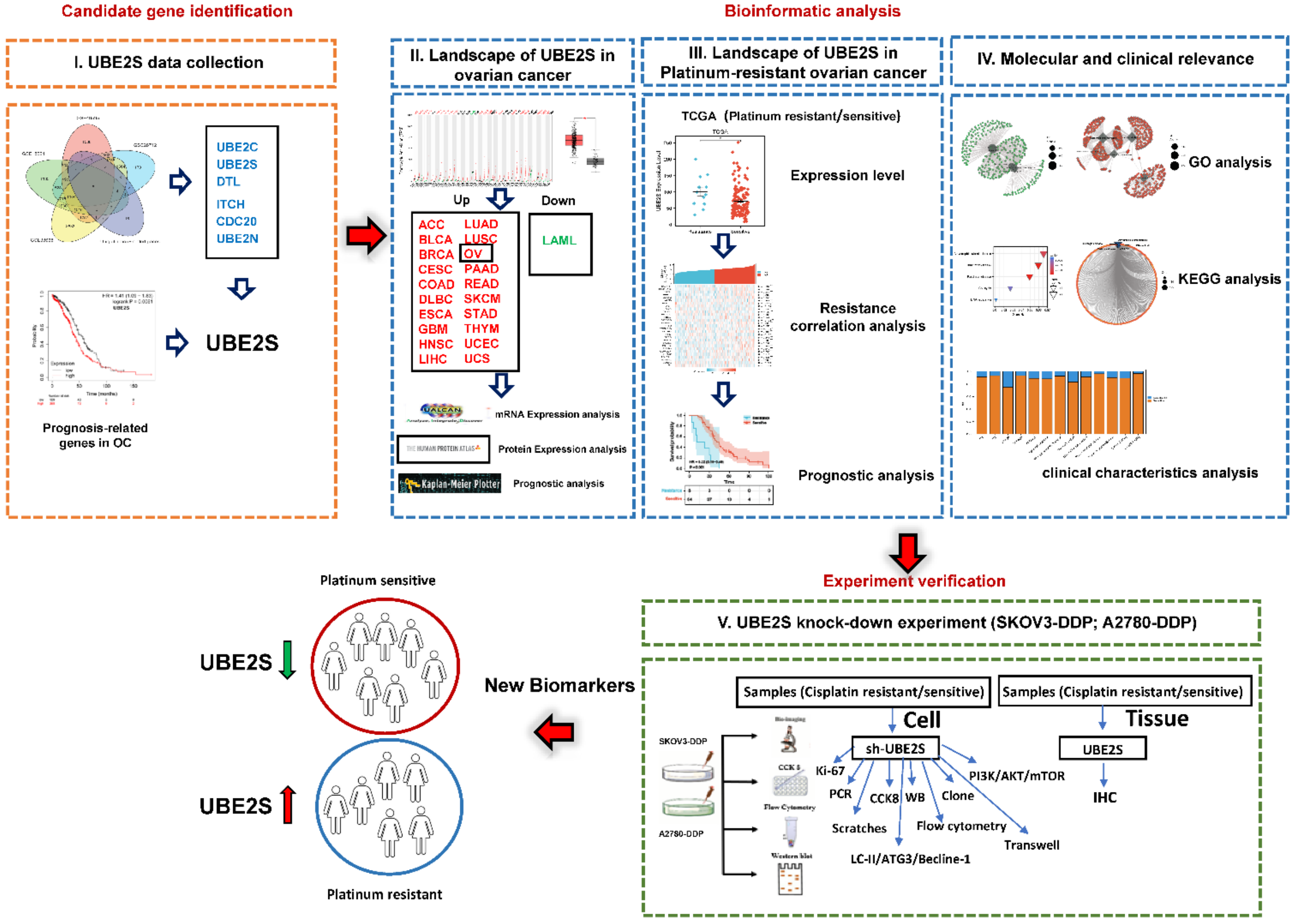

Candidate gene identification
Bioinformatic analysis
I. UBE2S data collection
II. Landscape of UBE2S in ovarian cancer
III. Landscape of UBE2S in Platinum-resistant ovarian cancer
IV. Molecular and clinical relevance
UBE2C
UBE2S
DTL
ITCH
CDC20
UBE2N
UBE2S
Prognosis-related genes in OC
Up
Down
ACC
LUAD
BLCA
LUSC
BRCA
OV
CESC
PAAD
COAD
READ
DLBC
SKCM
ESCA
STAD
GBM
THYM
HNSC
UCEC
LIHC
UCS
LAML
mRNA Expression analysis
Protein Expression analysis
Kaplan-Meier Plotter
Prognostic analysis
TCGA (Platinum resistant/sensitive)
Expression level
Resistance correlation analysis
Prognostic analysis
GO analysis
KEGG analysis
clinical characteristics analysis
Experiment verification
V. UBE2S knock-down experiment (SKOV3-DDP; A2780-DDP)
Samples (Cisplatin resistant/sensitive)
Cell
Samples (Cisplatin resistant/sensitive)
Tissue
SKOV3-DDP
A2780-DDP
sh-UBE2S
Ki-67
PCR
CCK8
WB
Clone
PI3K/AKT/mTOR
Scratches
Flow cytometry
Transwell
LC-II/ATG3/Becline-1
UBE2S
IHC
Platinum sensitive
UBE2S
New Biomarkers
UBE2S
Platinum resistant
