# Supplementary figures and images for: Activation of PI3K/AKT/mTOR signaling axis by UBE2S inhibits autophagy leading to cisplatin resistance in ovarian cancer
Source: J Ovarian Res. 2023 Dec 19;16:240. doi: 10.1186/s13048-023-01314-y (PMC10729389; doi:10.1186/s13048-023-01314-y)

A

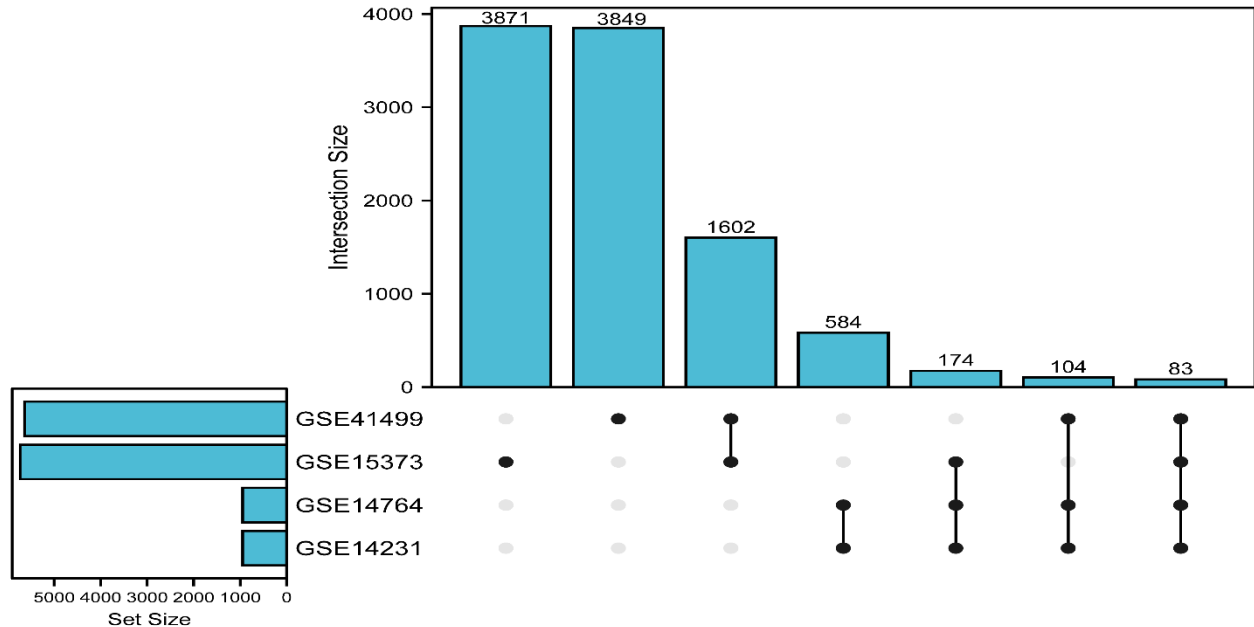

B

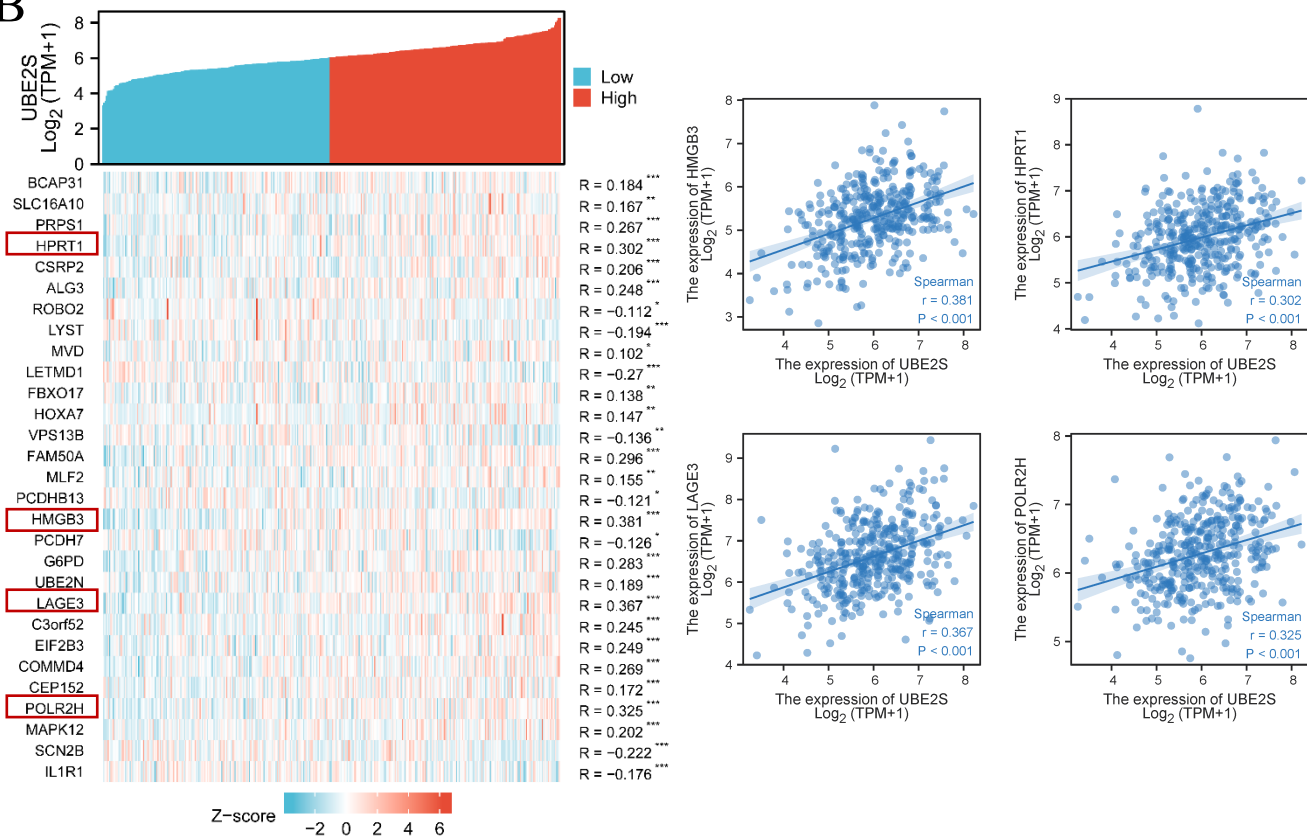

Supplement: Supplementary file 4 — Additional file 4: Figure S2. Correlation analysis between UBE2S and platinum resistance-related genes in patients with ovarian cancer. [file 13048_2023_1314_MOESM4_ESM.pdf]
