# Supplementary material for: Activation of PI3K/AKT/mTOR signaling axis by UBE2S inhibits autophagy leading to cisplatin resistance in ovarian cancer
Source: J Ovarian Res. 2023 Dec 19;16:240. doi: 10.1186/s13048-023-01314-y (PMC10729389; doi:10.1186/s13048-023-01314-y)
Supplement: Supplementary file 5 — Additional file 5: Figure S3. The effect of UBE2S gene knockdown on the sensitivity of ovarian cancer cells to cisplatin treatment and the construction of cisplatin-resistant cell lines. [file 13048_2023_1314_MOESM5_ESM.pdf]

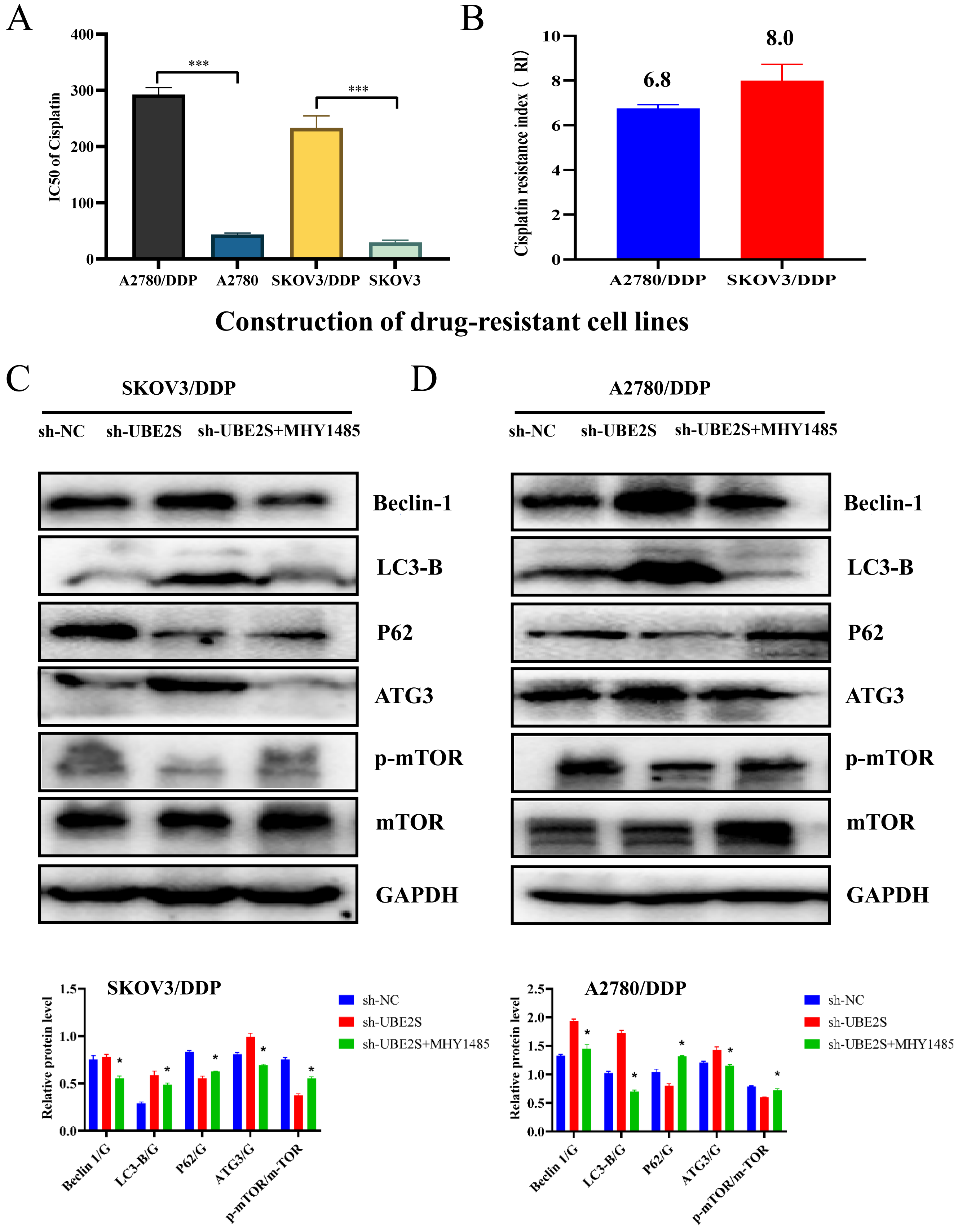
A
400
300
200
100
0
IC50 of Cisplatin
***
***
A2780/DDP
A2780
SKOV3/DDP
SKOV3
B
10
8
6
4
2
0
Cisplatin resistance index (RI)
6.8
8.0
A2780/DDP
SKOV3/DDP
Construction of drug-resistant cell lines
C
SKOV3/DDP
sh-NC
sh-UBE2S
sh-UBE2S+MHY1485
Beclin-1
LC3-B
P62
ATG3
p-mTOR
mTOR
GAPDH
D
A2780/DDP
sh-NC
sh-UBE2S
sh-UBE2S+MHY1485
Beclin-1
LC3-B
P62
ATG3
p-mTOR
mTOR
GAPDH
SKOV3/DDP
Relative protein level
1.5
1.0
0.5
0.0
sh-NC
sh-UBE2S
sh-UBE2S+MHY1485
Beclin 1/G
LC3-B/G
P62/G
ATG3/G
p-mTOR/m-TOR
A2780/DDP
Relative protein level
2.5
2.0
1.5
1.0
0.5
0.0
sh-NC
sh-UBE2S
sh-UBE2S+MHY1485
Beclin 1/G
LC3-B/G
P62/G
ATG3/G
p-mTOR/m-TOR
